# Characterizing Measurement Error in Dietary Sodium in Longitudinal Intervention Studies

**DOI:** 10.3389/fnut.2020.581439

**Published:** 2020-11-27

**Authors:** Adam Pittman, Elizabeth A. Stuart, Juned Siddique

**Affiliations:** ^1^Department of Biostatistics, Johns Hopkins Bloomberg School of Public Health, Baltimore, MD, United States; ^2^Department of Mental Health, Johns Hopkins Bloomberg School of Public Health, Baltimore, MD, United States; ^3^Department of Preventive Medicine, Northwestern University Feinberg School of Medicine, Chicago, IL, United States

**Keywords:** lifestyle intervention, measurement error, nutrition, sodium intake, biomarkers, self-reporting habits

## Abstract

**Background:** Previous measurement error work that investigates the relationship between a nutritional biomarker and self-reported intake levels has typically been at a single time point, in a single treatment group, or with respect to basic patient demographics. Few studies have examined the measurement error structure in longitudinal randomized trials, and whether the error varies across time or group. This structure is crucial to understand, however, in order to correct for measurement error in self-reported outcomes and properly interpret the longitudinal effects of dietary interventions.

**Methods:** Using two longitudinal randomized controlled trials with internal longitudinal validation data (urinary biomarkers and self-reported values), we examine the relationship between urinary sodium and self-reported sodium and whether this relationship changes as a function of time and/or treatment condition. We do this by building a mixed effects regression model, allowing for a flexible error variance-covariance structure, and testing all possible interactions between time, treatment condition, and self-reported intake.

**Results:** Using a backward selection approach, we arrived at the same final model for both validation data sets. We found no evidence that measurement error changes as a function of self-reported sodium. However, we did find evidence that urinary sodium can differ by time or treatment condition even when conditioning on self-reported values.

**Conclusion:** In longitudinal nutritional intervention trials it is possible that measurement error differs across time and treatment groups. It is important for researchers to consider this possibility and not just assume non-differential measurement error. Future studies should consider data collection strategies to account for the potential dynamic nature of measurement error, such as collecting internal validation data across time and treatment groups when possible.

## Introduction

Dietary interventions seek to change dietary behaviors – either to affect some clinical outcome or to change the behavior itself. These studies might use only one time point after baseline to assess participant outcomes, or they may be longitudinal, in which participant outcomes are measured several times over the course of months or years after initial group assignment.

Dietary intervention studies usually require investigators to collect nutrient intake data— such as sodium consumption in study participants—to estimate the effect of the intervention on diet. Yet properly measuring dietary intake, especially over time, with high accuracy can be difficult. Direct nutrient intake is rarely observed, and in dietary studies, researchers frequently resort to two methods to measure nutrient intake: self-report or biomarkers (1).

Self-reported measures generally rely on participants reporting their dietary intake over some period of time, such as the past 24 h or 7 days. This often takes the form of a food frequency questionnaire (FFQ), where participants fill out a survey about their eating habits or a 24-h dietary recall, where participants report everything consumed over the previous day. That is then used to extract information about the nutrients in the food reported as having been consumed. Biomarkers are biologic components from participants, such as blood, urine, or hair, which contain information about a person's nutrient levels. Biomarkers are useful because they objectively measure intake and some provide unbiased estimates of intake. Therefore, biomarkers may be closer to the “truth” than self-reported methods (but still subject to measurement error), and hence provide a better estimate of a person's nutrient intake (2, 3).

Unfortunately, biomarkers are often expensive, invasive, and/or difficult to implement in a study (4). They place potentially greater burden on study participants than self-report measures; this burden may discourage participants from taking part in a longitudinal study. Thus, there are concerns that biomarkers can contribute to poor study adherence and missing data problems (i.e., that participants will drop out of the study because of the hassle or invasiveness of the biomarker collection) (5). For these reasons, in many studies, it is often infeasible to capture biomarker data over time. Self-reported methods are more frequently implemented than biomarker measurements since they are likely easier, cheaper, and more convenient for the participant (4).

Both of these methods (biomarkers and self-report) act as “proxy” measurements of true intake, because they can be representative, but are potentially imprecise versions of the truth. They are potentially subject to two main types of error: systematic and random. Systematic error, or bias, means that a measure consistently departs from the truth in the same direction (i.e., always higher or lower), and can be hard to detect and analyze statistically (6). Systematic errors can decrease the accuracy of measurements and create potentially erroneous conclusions about the relationship between food intake or nutrients and nutrition-related diseases (7). Random error can create variability in the measurements, which may reduce precision, resulting in a loss of statistical power. However, random errors can be more easily corrected with statistical methodology (8). These errors together help create measurement error, the difference between “true” and “observed” intake.

If researchers are concerned with measurement error, they may have a slight preference for biomarker collection methods because the objective nature of biomarkers leads to less systematic error, but they are still subject to potential random errors such as daily variation in diet (3, 8–10). Self-reported measures can be more susceptible to systematic measurement errors due to the many complexities of properly reporting food intake (8, 11, 12). Even with the best due diligence, factors such as social desirability or recall problems influence final results. Examples include constant over or under-reporting (systematic error) or daily fluctuations in food consumption (random error).

Given these measurement challenges in nutrition (and many other fields), researchers have developed statistical methods such as *regression calibration* (13) and *Simulation Extrapolation* (SIMEX) (14) to deal with measurement error in settings where the variable measured with error is a covariate in an outcome model. To implement these methods, it is necessary to have information on the relationship between the variable measured with error and its true value.

The existing measurement error literature in dietary studies, and their respective correction methods, typically examine measurement error at one specific time point and/or in a single observational cohort. However, these measurement error patterns may not remain constant in longitudinal lifestyle interventions.

In addition, in randomized controlled trials (RCTs), where individuals are randomly assigned to treatment conditions and the intervention and comparison groups have different experiences, self-reporting behaviors could change over time and/or by treatment assignment. Those in the treatment group may become more cognizant of nutrition intake through intervention exposure, leading to increased reporting accuracy. Participants may also modify their self-reported values (even if not necessarily their true intake) to appear compliant with intervention recommendations, which decreases their accuracy (12).

Self-reported precision could also wane over time as participants experience fatigue with repeated reporting (15). This fatigue could lead them to be more carefree and less rigorous, introducing uncertainty into measurements. Conversely, as people repeatedly monitor sodium intake over time, they may become more accurate with increased repetitions. Thus, the structure of the measurement error could be *differential*, meaning the amount of error may differ across treatment groups and could change over time differently for each treatment group. However, to this point there has been little empirical investigation of these patterns.

As a case study, we examined sodium intake in two longitudinal intervention trials, Trials of Hypertension Prevention (TOHP) (16) and PREMIER: Lifestyle Interventions for Blood Pressure Control (17). These data sets are particularly useful for examining measurement error over time because they, unlike most dietary intervention trials, contain both self-reported sodium intake via 24-h recall and a sodium biomarker−24-h urine—for each participant at every time point. With this information, we compare the participants' self-reported values with their directly measured urinary sodium to characterize the measurement error, and assess whether the error varies across treatment group and time. Our analyses could be helpful to learn about potential measurement error in other settings, and to help researchers understand when it is important to consider differential measurement error by time or treatment condition.

## Materials and Methods

### Trials of Hypertension Prevention

TOHP was a U.S. based, multicenter, randomized trial of 2,182 participants testing the efficacy of a lifestyle intervention aimed at lowering diastolic blood pressure (DBP) from the high normal range (80–89 mmHg) (16) to a lower range. Participants were assigned to one of four treatment groups: sodium reduction, weight reduction, stress management, or control. The sodium reduction group received counseling on how to reduce sodium consumption in everyday life. The weight reduction group received guidance on weight-loss techniques. The stress management group were provided coping mechanisms to handle stressful situations. The weight loss and stress management groups did not receive any counseling specifically on sodium intake. The control group did not receive any particular intervention or information; in this sense it was similar to a “usual care” condition.

Participants were considered eligible if they were healthy men and women, aged 30 through 54 years, who had high normal DBP and were not taking antihypertensive drugs for the prior 2 months (16). All participants were screened three times prior to enrollment to check eligibility requirements and then randomized to one of the four treatment groups. On the third screening, a 24-h dietary recall was conducted, and participants provided a 24-h urine sample; this served as their “baseline” measurement. All participants were contacted again—at an unannounced point in time— ~6 and 18 months after enrollment to again provide 24-h dietary recall and 24-h urine biomarker for sodium consumption at each respective time point (Table 1). The 24-h recall data on individual foods was converted into nutrients using the Tufts Nutrient Data Bank based on the US Department of Agriculture Standard Reference (Release 9) in combination with extensive manufacturers' data and published nutrient data on currently consumed food products (16, 18, 19).

**Table 1:** Study characteristics and participant demographics in TOPH and PREMIER studies.

	**TOHP^†^**	**PREMIER**
*N*	751	818
Enrollment dates	1988–1990	1999–2001
Timing of sodium assessment	Baseline	Baseline
	6 months	6 months
	18 months	18 months
Assessment method	24-h recall	Two 24-h recalls
	24-h urine	24-h urine
Treatment categories (*N* in group)	Sodium reduction^*^ (329) Control‘ (422)	Established^*^ (271) Established plus DASH^*^ (272) Advice only‘ (275)
Male N (%)	534 (71)	310 (38)
Mean baseline BMI (SD)	27.3 (3.6)	33.2 (5.7)
Mean baseline age (SD)	43 (6.4)	50 (8.9)

† *Some cohorts in the original TOHP study were outside the scope of our analysis and therefore were excluded from the final models. N = 751 reflects a subset of TOHP we used for this study, and subsequent BMI and age calculations are derived from the subsetted population*.

* *Categorized as “treatment” for our purposes*.

*‘Categorized as “control” for our purposes*.

### PREMIER: Lifestyle Interventions for Blood Pressure Control

PREMIER was also a U.S. based, multicenter randomized trial testing the effects of various lifestyle intervention on blood pressure outcomes in 810 adults with above optimal DBP (80–95 mmHg) and who were not taking antihypertensive medications (17).

Participants were randomly assigned to one of three treatment groups: Established, Established Plus Dash, or Advice Only. The Established group received guidance on improving their dietary habits (including reducing sodium consumption) and increasing physical activity. Established Plus Dash received an intervention similar to Established but also received education on the DASH diet, a diet high in fruits, vegetables and low-fat dairy products. Finally, Advice Only received general healthy behavior advice, but no specific counseling on sodium intake or physical activity.

All eligible participants attended a randomization visit, where researchers randomized them to a group and then collected baseline measurements including two 24-h dietary recalls, and a 24-h urine sample. Trial researchers contacted all participants unannounced at 6 and 18 months after enrollment, at which point individuals again provided two 24-h dietary recalls and 24-h urine samples (Table 1).

Intake of nutrients and food groups was assessed from unannounced 24-h dietary recalls conducted by telephone interviewers. Two recalls (one obtained on a weekday and the other on a weekend day) were obtained at baseline, 6-, and 18-months by the Diet Assessment Center of Pennsylvania State University. The Nutrition Data System (NDS) developed and maintained by the Nutrition Coding Center of the University of Minnesota was used to generate the estimates of individual nutrient intake from the recalls (17).

We obtained the datasets for TOHP and PREMIER through an online request from the National Heart, Lung, and Blood Institute BioLINCC data repository after receiving IRB approval through Johns Hopkins Bloomberg School of Public Health and Northwestern University.

For both datasets we consolidated the original treatment and control groups into new ones for our purposes. In TOHP, only the sodium reduction group received counseling on sodium management. Hence, we discarded the stress management and weight reduction groups and only use the original control group in the control arm. For the PREMIER study we considered both behavioral intervention groups (Established, Established plus DASH) as the “treatment” condition, and used the advice only condition as the control condition. We are interested in whether participants in the sodium reduction interventions, more (or less) accurately report their actual sodium intake compared to those in the advice only group, and whether the pattern of measurement error varies over time.

The same data cleaning procedures were used for both studies prior to analysis. First, the biomarker sodium values were converted to dietary sodium values by dividing urine sodium values by 0.86, as only 86% of sodium intake appears in urine (1, 20). The dietary sodium and self-reported sodium values were both natural log-transformed to make the respective distributions approximately normal. In PREMIER, the two self-reported sodium values at each time point were averaged after log transformation. We centered log self-report (log self-report – mean log self-report at baseline) to help with the interpretability of regression coefficients.

Our model of interest is a calibration model in which a reference measure (urinary sodium) is regressed on its self-reported version (21). This relationship is used for missing data approaches (22) for handling measurement error where the variable measured without error is treated as missing data and imputation is used to fill in the unobserved data (23–26).

We began by plotting the data in order to visualize the relationship between urinary sodium and self-reported sodium and help inform our modeling efforts. We used scatterplots of urinary sodium against self-reported sodium, grouped by time, with an overlapping linear predicted regression line for each condition at each time point.

Mixed effects linear regression was used (27) to estimate the relationship between log measured urinary sodium and log self-reported sodium over time, and by treatment group, while taking into account the correlation of measures within a participant over time. To estimate these models, we used the lme4 and lmerTest packages in R version 3.5.1 (28–30).

For each trial, we started with an initial model that included main effects for follow-up time (indicators for 6- and 18-months), subjects' self-reported intake, as well as two-way interactions between self-reported intake and time, time and treatment assignment, and a three-way interaction between self-reported intake, time, and treatment. We allow each individual to have a random intercept, and the (log centered) self-reported values to have a random slope, and used an unstructured covariance matrix to model the random effects.

For each person *i* (*i* = 1,…, *N*), at time *j* (*j* = baseline, 6 months, 18 months; coded categorically), in our defined treatment group (TX; 0 = control, 1 = treatment) their urine measured sodium intake is represented by *U*_*ij*_ and self-reported intake is represented by *self*_*ij*_. Our model can be written as:

(1) $$\begin{array}{l} U_{ij}=\beta_{0}+\beta_{1}*self_{ij}+\beta_{2}I\left(time_{j}=6\right)+\beta_{3}I\left(time_{j}=18\right)\\ \;+\;\beta_{4}I\left(time_{j}=6\right)*TX_{i}+\;{\;\beta }_{5}I\left(time_{j}=18\right)\\ \;*TX_{i}+\;\beta_{6}*self_{ij}I\left(time_{j}=6\right)+\;\beta_{7}*self_{ij}I\left(time_{j}=18\right)\\ \;+\;\beta_{8}*self_{ij}I\left(time_{j}=6\right)*TX_{i}+\beta_{9}*self_{ij}I\left(time_{j}=18\right)\\ \;*TX_{i}+\;b_{0i}+\;b_{1i}*self_{ij}+e_{ij} \end{array}$$

In Equation (1) *I*() is an indicator function which takes on either 0 or 1. *b*_0*i*_ is the random intercept and *b*_1*i*_ is the random slope for each person's centered self-reported values, respectively. We assume correlated random effects where *b*_0*i*_ ~ *N* (0, $\tau_{0}^{2}$), *b*_1*i*_ ~*N* (0, $\tau_{1}^{2}$), and residual error terms $e_{ij}\sim N\left(0,\;\sigma^{2}\right)$, independent of the random effects

We excluded a main effect for treatment (TX) from the model because the coefficient was ~0. This is expected because we assume treatment and control groups have similar sodium levels at baseline, at least in expectation (because of randomization) and thus reduces an extra parameter.

Including the three-way (self-reported intake by time by treatment) interactions in this initial model allows the relationship between urinary sodium and self-reported sodium to vary over time and across the treatment and control groups. We include a time by treatment interaction to examine whether average levels of urinary sodium differ by time and treatment condition at a fixed level of self-report.

A backwards variable selection approach was used to obtain a final analysis model. First, the initial saturated model with the three-way interaction shown in Equation (1) was fit. We used a significance level of 0.2 to decide whether a variable should remain in the model. We first tested the two three-way interactions self-report^*^time^*^treatment. If at least one coefficient had a *p*-value < 0.2, we kept both interaction terms in the model (i.e., for both time points). If both coefficients had *p*-value > 0.2, we dropped them from the model and refit our second-stage model which omits the 3-way interaction.

In our second-stage model, we tested the significance of the self-report^*^time terms (β_6_, β_7_), which measure whether the relationship between urinary sodium and self-reported sodium changes over time, assuming any change is constant across the treatment and control groups. Once again, if both coefficients had *p*-values > 0.2, we dropped them from the model and fitted our final model.

Our final model allows urinary sodium levels to change across time and treatment status. In this model we test the time^*^treatment interactions (β_4_, β_5_). If both coefficients had *p*-values > 0.2, we dropped them from the model.

After selecting our final model we then standardized the regression coefficients. To standardize the exposure—self-reported intake—we subtracted the pooled (control and treatment) mean self-reported intake at baseline from all self-reported values and then divided that result by the standard deviation of self-reported intake at baseline. The outcome—urinary sodium—was similarly standardized, using the pooled mean and standard deviation of urinary sodium at baseline.

## Results

**Figure 1:**
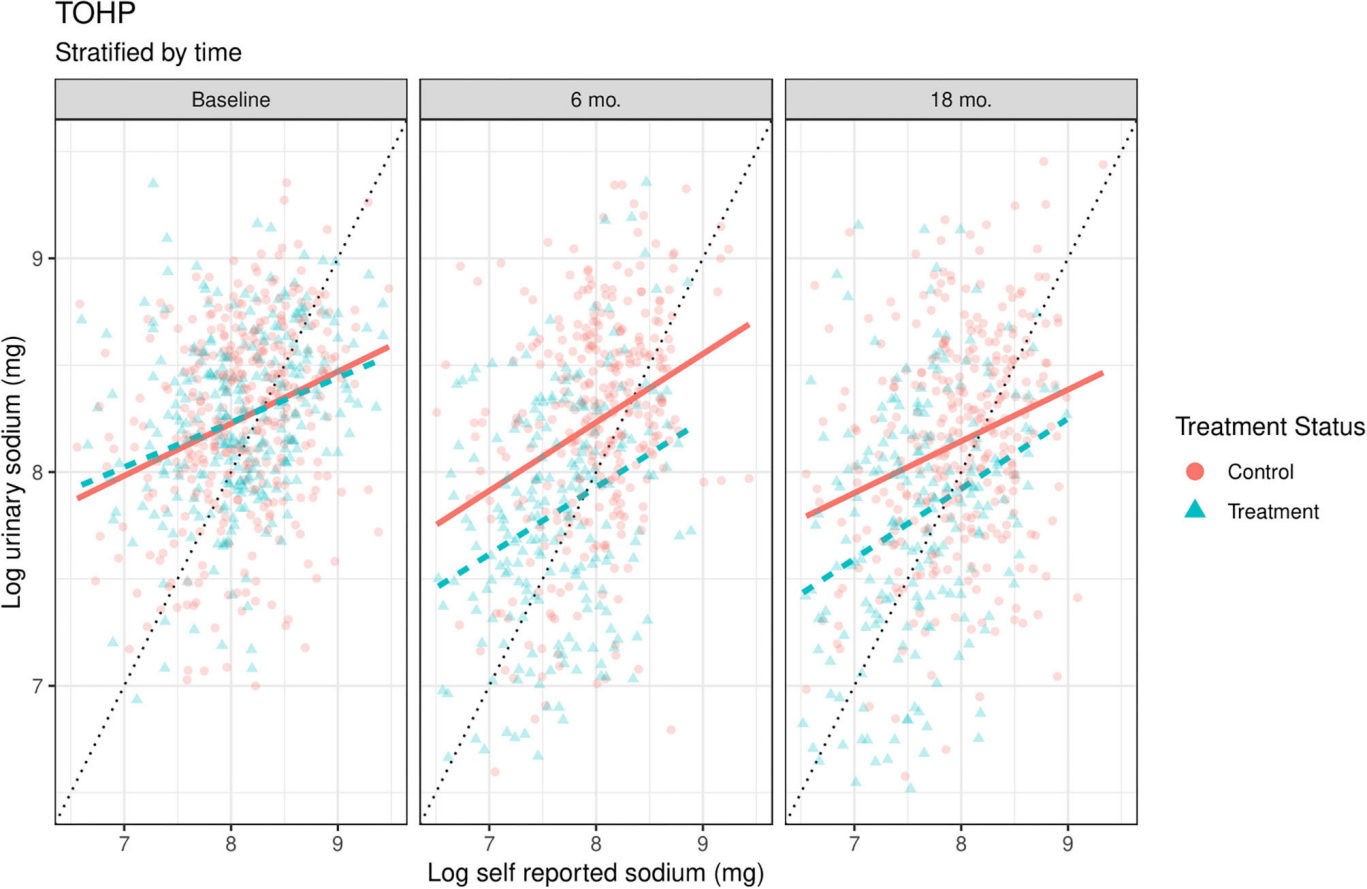
Scatterplots of log urinary sodium vs. log self-reported sodium by time and treatment conditions in TOHP. The solid orange (control) and dashed blue lines (treatment) are linear smoothers of urinary sodium as a function of self-reported sodium in each treatment condition. 45-degree line represents where urinary sodium equals self-reported sodium. Units are on the natural log scale.

**Figure 2:**
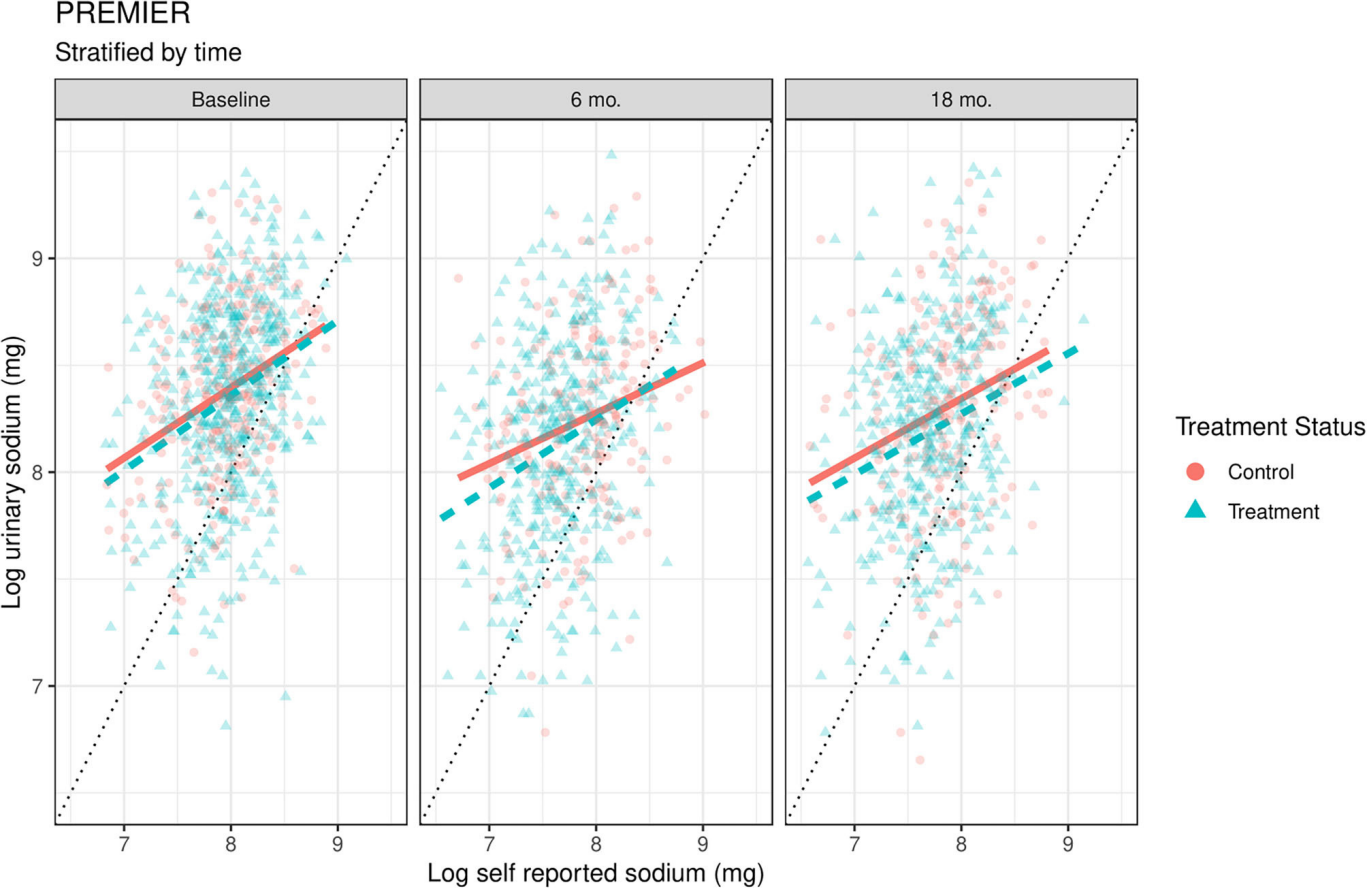
Scatterplots of log urinary sodium vs. log self-reported sodium data time and treatment conditions in PREMIER. The solid orange (control) and dashed blue lines (treatment) are linear smoothers of urinary sodium as a function of self-reported sodium in each treatment condition. 45-degree line represents where urinary sodium equals self-reported sodium. Units are on the natural log scale.

Both datasets include people who over and under report by time and treatment status (Figures 1, 2). The 45-degree line in each graph represents “perfect” reporting, where measured urine biomarker equals self-reported sodium. Those who fall above the line under report, meaning their measured urine sodium levels were higher than self-reported intake. Conversely, those below the line over report, meaning their measured urine sodium levels were lower than their self-reported amounts. The wide scattering of points suggests a high degree of variability in reported sodium levels.

We overlapped a linear smoother on top of the scatterplot to highlight some reporting differences between the treatment and control conditions. These lines should be considered as preliminary models, as they fit the models separately by time and group, and thus do not allow formal model comparisons across time or group, but the relationships between self-reported and biomarker values appear broadly similar. In both studies at baseline, the two study conditions are approximately equal in urinary vs. dietary sodium levels, as expected from the randomization.

### Regression Results

Using the stepwise procedure described above, neither the three-way interactions in model (**1**), nor the interactions between self-reported sodium levels and time in the second-stage model met the criteria for inclusion in either study. As such, the final model for both studies only includes the interaction between treatment and time. This final model is shown in Equation (2).

(2) $$\begin{array}{l} U_{ij}=\beta_{0}+\beta_{1}*self_{ij}+\beta_{2}I\left(time_{j}=6\right)+\beta_{3}I\left(time_{j}=18\right)\\ \;+\;\beta_{4}I\left(time_{j}=6\right)*TX_{i}+{\;\beta }_{5}I\left(time_{j}=18\right)*TX_{i}\\ \;+\;b_{0i}+\;b_{1i}*self_{ij}\;+e_{ij} \end{array}$$

This model implies that average measured urinary sodium changes over time (β_2_, β_3_), and at different rates in the treatment group vs. control group (β_4_, β_5_) but that there is no differential change in the slope of self-reported sodium across groups over time. It is interesting to note that the final regression results in both datasets were very similar to one another.

In TOHP (Table 2), there was a small but significant decrease in urinary sodium between baseline and 18 months in the control group. The control group at 18 months has 0.19 SD lower urinary sodium than the control group at baseline on the log scale (β_3_ = −0.19). There was, on average, a much larger significant decrease in measured urine sodium between baseline and each follow up time for the treatment group, for a given level of self-reported sodium. At 6 months, the treatment group has 0.81 SD lower urinary sodium than control group (β_4_ = −0.81), and 0.65 SD lower at 18 months on the log scale (β_4_ = −0.81).

**Table 2:** Standardized regression output from the final regression model.

**Parameter from model (2)**	**TOHP**		**PREMIER**	
	**Estimate (95%CI)**	***p*-value**	**Estimate (95%CI)**	***p*-value**
Centered self-report β_1_	0.29 (0.23, 0.34)	<0.001	0.21 (0.17, 0.26)	<0.001
Month 6 control β_2_	0.03 (−0.10, 0.16)	0.68	−0.24 (−0.37, −0.10)	<0.001
Month 18 control β_3_	−0.19 (−0.32, −0.06)	0.005	−0.08 (−0.21, 0.05)	0.23
Month 6 Trt. β_4_	−0.81 (−1.0, −0.63)	<0.001	-0.1 (−0.26, 0.06)	0.20
Month 18 Trt. β_5_	−0.65 (−0.84, −0.47)	<0.001	−0.15 (−0.30, 0.0)	0.06

*β_1_: Change in average log urinary sodium (in SD units) due to a 1 SD unit change in log self-reported sodium at baseline for treatment and control (assumed to be same across groups at baseline because of randomization)*.

*β_2_: Among the control group members, difference in average urinary sodium (in SD units) between baseline and 6 months on the log scale*.

*β_3_: Among the control group members, difference in average urinary sodium (in SD units) between baseline and 18 months on the log scale*.

*β_4_: Difference in average urinary sodium (in SD units) between treatment and control groups at 6 months on the log scale*.

*β_5_: Difference in average urinary sodium (in SD units) between treatment and control groups at 18 months on the log scale*.

In PREMIER (Table 2), there was a significant decrease in average measured urine sodium at 6 months compared to baseline. Both groups at 6 months had 0.24 SD lower urinary sodium at baseline on the log scale (β_2_ = −0.24). However, this difference was no longer there at 18 months. There were no significant difference between treatment and control groups at any point in PREMIER.

If the relationship between urinary sodium and self-reported sodium did not change over time and by treatment condition, we would expect β_2_, β_3_, β_4_, β_5_ = 0. Instead, we find that β_2_, β_3_, β_4_, β_5_ < 0, an indication that the relationship between urinary sodium and self-reported sodium does in fact change over time and by treatment status. In general, for a given level of self-report, urinary sodium is *lower* at follow-up than it is at baseline.

## Discussion

We expand on the current nutrition literature by focusing on the differential measurement error structure of self-reported intake which may arise when the treatment group self-reports their sodium intake with increased or decreased accuracy (31). We do this by modeling the relationship of urinary sodium as a function of self-reported sodium, time, treatment condition and all possible interactions. This information is important when designing studies where self-reported intake is a longitudinal outcome variable, and can help inform measurement error correction methods that use missing data approaches to correct for measurement error.

The final models for TOHP and PREMIER look very similar to one another, with slightly different coefficient values. The slopes of self-reported sodium did not change as a function of time or by treatment condition. The lack of significance in the three-way self-report^*^time^*^treatment interaction and the two-way self-report^*^time interaction indicates a lack of significant difference in systematic error in terms of the relationship between self-reported sodium and urinary sodium between the treatment arms across all three time points. However, the intercepts do change by time and/or treatment condition indicating that measurement error is affected by time and/or treatment condition. Further, our final models were much more parsimonious than our initial, fully saturated model. This result suggests that relatively simple measurement error correction models that involve only shifts in the intercept of the calibration model are sufficient to appropriately correct for measurement error.

In PREMIER, we see a decrease in measured urine sodium—conditioning on self-report—at 6 months in the control group, whereas in TOHP we see a much stronger decrease in the treatment group at 6 and 18 months. These results suggest that the relationship between biomarker and self-report can differ by treatment group and/or time, however, these differences may be study specific.

A failure to take into account differential measurement error could result in biased estimates of the treatment effect. For example, in TOHP at 6-months, for a given level of self-reported sodium, participants in the treatment condition had lower urinary sodium than did control participants. A measurement error correction model that did not take this difference into account would result in an attenuated treatment effect because this difference in reporting would not be incorporated into the difference between groups.

Discrepancies in the literature still exist about the relationship between treatment and self-reporting error. Other studies have found evidence for a relationship between treatment assignment and self-report bias, similar to the results of TOHP. In the Women's Health Eating and Living Study, a longitudinal randomized intervention trial with validation data (32), researchers found dietary intervention affected measurement error in self-reported outcomes using plasma carotenoid biomarkers. In the Women's Health Initiative Dietary Modification Trial, another dietary intervention trial (33), participants in the control group under-reported protein intake at greater amounts compared to the treatment arm. There is thus evidence that there may be differential measurement error across time and treatment group, and that this may vary depending on the dietary component being measured.

One possible solution to examine and address measurement error across time and treatment groups would be internal validation datasets with longitudinal intervention aspects. While this route is resource intensive, it may be worthwhile if it allows researchers to estimate treatment effects with less bias and greater power to detect significant effects. A cheaper or less invasive biomarker would make creating this dataset more feasible. Another option would be more measurement error correction methods, which is why it is important to study how measurement error structures change over time and by treatment status. Siddique et al. (25) performed sensitivity analyses to the assumption that measurement error structure is time invariant, treatment invariant, and time and treatment invariant. Understanding how measurement error changes over time and by treatment condition in validation datasets can help encourage the implementation of these methods and improve the accuracy of self-reported measures in longitudinal intervention trials without available biomarker data.

### Limitations

One limitation of this study is the amount of missing data, with the highest being 29% at 18 months in TOHP and the lowest being 1% at baseline in both studies. The regression models were fit assuming that the missing data was “missing at random” (MAR). This means we assume participants with unobserved dietary sodium information at a given time point will have similar intake values as the observed participants at the same time after conditioning on other observed values (34). This assumption may not hold in all circumstances however, and if violated could imply differences between the observed and unobserved groups. Future work could examine how the patterns of missingness may interact with measurement error structures.

In both studies, the 24-h recalls and the 24-urine samples were not required to capture the same day of measurement. We assume that these two measures are capturing estimates of short-term intake. Even so, the limited number of measurements at each time point is likely not adequate to capture usual intake. Estimates from both the biomarker and self-reported data are therefore subject to additional variability due to day-to-day variation in diet (1).

The biomarker sodium levels—measured through urine—are also subject to additional sources of variability. Urinary sodium excretion may reflect more than 1 day of intake (35). Further, we divided urinary sodium values by 0.86 under the assumption that 86% of consumed sodium is available in urine (1, 20), this value is likely to differ by participant, introducing additional uncertainty in our estimates (36). These sources of variability in 24-h recalls and urinary sodium would have the result of attenuating the relationship between self-reported sodium and urinary sodium in our models.

### Conclusion

We found that the measurement error structure in longitudinal studies can differ by time and treatment condition. When correcting for measurement error, intervention researchers need to take these differences into account, either by designing internal validation studies that are also longitudinal or by implementing measurement error correction methods that are explicitly designed to account for these changes in measurement error. Lifestyle intervention trials that fail to do this may draw erroneous conclusions of their results.

## Data Availability Statement

Publicly available datasets were analyzed in this study. This data can be found here: https://biolincc.nhlbi.nih.gov/studies/premier/, https://biolincc.nhlbi.nih.gov/studies/tohp/.

## Ethics Statement

The studies involving human participants were reviewed and approved by Johns Hopkins Bloomberg School of Public Health IRB Northwestern University IRB. The patients/participants provided their written informed consent to participate in this study.

## Author Contributions

JS originally conceived the idea for studying measurement error in longitudinal studies, and created the original model. AP performed the analyses and drafted the manuscript. ES provided assistance on analyses. All authors contributed to the article and approved the submitted version.

## Conflict of Interest

The authors declare that the research was conducted in the absence of any commercial or financial relationships that could be construed as a potential conflict of interest.
